# Assessment of a Simulated Case-Based Measurement of Physician Diagnostic Performance

**DOI:** 10.1001/jamanetworkopen.2018.7006

**Published:** 2019-01-11

**Authors:** Souvik Chatterjee, Sanjay Desai, Reza Manesh, Junfeng Sun, Shantanu Nundy, Scott M. Wright

**Affiliations:** 1 Critical Care Medicine Department, Medstar Washington Hospital Center, Washington, DC; 2 Department of Medicine, Johns Hopkins University School of Medicine, Baltimore, Maryland; 3 Critical Care Medicine Department, Clinical Center, National Institutes of Health, Bethesda, Maryland; 4 The Human Diagnosis Project, Washington, DC; 5 Milken Institute School of Public Health, George Washington University, Washington, DC; 6 Division of General Internal Medicine, Johns Hopkins Bayview Medical Center, Johns Hopkins University School of Medicine, Baltimore, Maryland

## Abstract

**Importance:**

Diagnostic acumen is a fundamental skill in the practice of medicine. Scalable, practical, and objective tools to assess diagnostic performance are lacking.

**Objective:**

To validate a new method of assessing diagnostic performance that uses automated techniques to assess physicians’ diagnostic performance on brief, open-ended case simulations.

**Design, Setting, and Participants:**

Retrospective cohort study of 11 023 unique attempts to solve case simulations on an online software platform, The Human Diagnosis Project (Human Dx). A total of 1738 practicing physicians, residents (internal medicine, family medicine, and emergency medicine), and medical students throughout the United States voluntarily used Human Dx software between January 21, 2016, and January 15, 2017.

**Main Outcomes and Measures:**

Internal structure validity was assessed by 3 measures of diagnostic performance: accuracy, efficiency, and a combined score (Diagnostic Acumen Precision Performance [DAPP]). These were each analyzed by level of training. Association with other variables’ validity evidence was evaluated by correlating diagnostic performance and affiliation with an institution ranked in the top 25 medical schools by *US News and World Report*.

**Results:**

Data were analyzed for 239 attending physicians, 926 resident physicians, 347 intern physicians, and 226 medical students. Attending physicians had higher mean accuracy scores than medical students (difference, 8.1; 95% CI, 4.2-12.0; *P* < .001), as did residents (difference, 8.0; 95% CI, 4.8-11.2; *P* < .001) and interns (difference, 5.9; 95% CI, 2.3-9.6; *P* < .001). Attending physicians had higher mean efficiency compared with residents (difference, 4.8; 95% CI, 1.8-7.8; *P* < .001), interns (difference, 5.0; 95% CI, 1.5-8.4; *P* = .001), and medical students (difference, 5.4; 95% CI, 1.4-9.3; *P* = .003). Attending physicians also had significantly higher mean DAPP scores than residents (difference, 2.6; 95% CI, 0.0-5.2; *P* = .05), interns (difference, 3.6; 95% CI, 0.6-6.6; *P* = .01), and medical students (difference, 6.7; 95% CI, 3.3-10.2; *P* < .001). Attending physicians affiliated with a *US News and World Report*–ranked institution had higher mean DAPP scores compared with nonaffiliated attending physicians (80 [95% CI, 77-83] vs 72 [95% CI, 70-74], respectively; *P* < .001). Resident physicians affiliated with an institution ranked in the top 25 medical schools by *US News and World Report* also had higher mean DAPP scores compared with nonaffiliated peers (75 [95% CI, 73-77] vs 71 [95% CI, 69-72], respectively; *P* < .001).

**Conclusions and Relevance:**

The data suggest that diagnostic performance is higher in those with more training and that DAPP scores may be a valid measure to appraise diagnostic performance. This diagnostic assessment tool allows individuals to receive immediate feedback on performance through an openly accessible online platform.

## Key Points

**Question:**

Can automated scoring by an online case-based simulator be used as a valid measure of diagnostic performance?

**Findings:**

This cohort study found that health care professionals with more experience and training demonstrated higher diagnostic performance scores, as measured on an online case simulator, The Human Diagnosis Project (Human Dx). Attending physicians were most efficient and accurate in diagnostic performance compared with residents, interns, and medical students.

**Meaning:**

Online case-based physician performance measurement has the potential to be a practical and scalable method in the assessment of diagnostic performance.

## Teaser

This cohort study evaluates the ability of an online case-based simulator to measure the diagnostic performance of physicians with varying levels of training and medical students.

## Introduction

The Institute of Medicine, in its 2015 report *Improving Diagnosis in Health Care,*^1^ estimated that most people will experience at least 1 diagnostic error in their lifetime and showed that “getting the diagnosis right” is a crucial component of effective health care. Autopsy, in-hospital adverse event monitoring, and malpractice claim data all demonstrate unacceptably high rates of diagnostic errors, often resulting in significant morbidity and mortality.^2,3,4^ Accrediting bodies, such as the Accreditation Council for Graduate Medical Education and the American Board of Internal Medicine, emphasize the foundational importance of diagnosis and clinical reasoning; these are intimately related to the core competencies of medical knowledge and patient care.^5^

Foundational research on clinical reasoning found that no single superior reasoning strategy differentiated novice from expert clinicians.^6^ Diagnostic expertise likely requires distinction in multiple cognitive domains: illness scripts and pattern recognition, decision trees, Bayesian reasoning, basic science, and physiology knowledge.^7^ Possibly because of the multidimensional cognitive approach required for expert diagnostic performance, in both undergraduate and graduate medical education, diagnostic performance is typically inferred rather than measured; impressions of evaluating faculty are based on direct, and more often indirect, supervision of clinical behavior.^8,9^ These observations are necessarily subjective and influenced by myriad biases including context and content specificity, frame of reference and personal characteristics of the observer, personal knowledge, and experience.^10^

As defined by the Institute of Medicine, diagnostic error is a “failure to establish an accurate and timely explanation of the patient’s health problem…,”^1^ so a combination of accuracy and efficiency can be considered the hallmark of optimal diagnostic performance. An ideal objective measure of diagnostic performance would (1) incorporate these 2 foundational components of diagnosis (accuracy and efficiency), (2) generate an assessment in real time with immediate feedback, (3) span a variety of content and contexts, and (4) be valid across experience levels, from students to practicing physicians. Case simulations that reveal information sequentially have the potential to measure both accuracy and efficiency, as they can assess not only whether the final diagnosis is correct (accuracy) but also whether the clinician is able to arrive at the correct diagnosis with less information (efficiency) (Figure). We conducted a study to validate the diagnostic performance scores derived from The Human Diagnosis Project (Human Dx).

**Figure.**
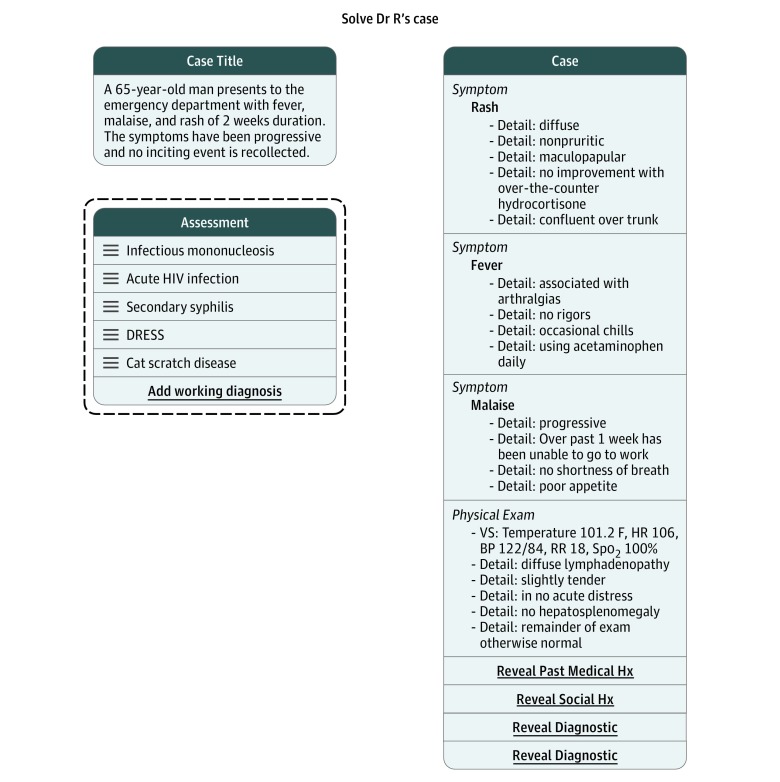
Representative Case From The Human Diagnosis Project Platform A case simulation on The Human Diagnosis Project includes a brief initial description in the case title and initial features of the presentation, listed under “case.” A differential diagnosis is free texted by the user into the “assessment” box. There are also several findings that can be revealed with mouse clicks: “reveal past medical Hx,” “reveal social Hx,” and “reveal diagnostic.” The differential diagnosis can then be reordered using the ladder next to the text and added to using the “add working diagnosis” feature. BP indicates blood pressure; DRESS, drug reaction with eosinophilia and systemic symptoms; HR, heart rate; Hx, history; RR, respiratory rate; Spo_2_, peripheral capillary oxygen saturation; VS, vital signs.

## Methods

### Design and Participants

This retrospective cohort study used data from individuals who are registered with Human Dx. Most users are attending physicians, residents, and medical students. For this study, international users were not included, as nomenclature of training level can vary. We also excluded residents and attending physicians from fields other than internal medicine, family medicine, and emergency medicine.

Basic access to Human Dx is free to any medical professional; upon registration, users create a profile in which they self-report their name, specialty, training level, and institutional affiliation. Use of deidentified user data for research purposes is part of Human Dx’s terms of service. Additional information about Human Dx can be found in the eAppendix in the Supplement. This study was approved by the Johns Hopkins University institutional review board.

### Cases and Data Used in the Analysis

Cases analyzed for this study were selected from the Global Morning Report (GMR) series within Human Dx. The GMR cases are the highest solver-rated cases submitted by users (all scored >9.0 on the 10-point quality scale). All GMR cases are peer reviewed by a member of the independent editorial board, which is composed of attending physicians at academic medical institutions with expertise in medical education. Cases cover a range of inpatient and outpatient presentations from general adult medicine and subspecialty disciplines. Case analyses were completed on all GMR cases solved from January 21, 2016, through January 15, 2017.

To allow for solver familiarity with the Human Dx scoring system and a technology learning curve, solvers with fewer than 2 case solves were excluded from analysis. Furthermore, only credible solve attempts were used in our analyses by excluding solves in which there was no attempt to create a differential diagnosis prior to revealing the last finding (1% of solves).

The raw data collected on each case solve include the ranked differential diagnosis at each step of the case mapped to major medical ontologies including the World Health Organization’s *International Classification of Diseases, Tenth Revision, Clinical Modification*, Unified Medical Language System, and Clinical Classifications Software. Scoring considers the ranked position of each diagnostic consideration listed and at what step in the case it was added or removed.

### Diagnostic Performance Outcomes

Two key metrics of diagnostic performance were analyzed for each user relative to other solvers on the same case: efficiency, a percentile score calculated based on the proportion of findings revealed before the user first includes the correct diagnosis in his or her differential diagnosis; and accuracy, analyzed 2 ways: (1) a percentile calculated from how high on the differential the correct diagnosis was at the end of the case and (2) a binary measure calculated with credit given for having the correct diagnosis in the first position of the user’s differential diagnosis at the end of the case (eg, with all findings revealed).

Because high efficiency can be achieved with low accuracy (thinking of a diagnosis with less information but not putting it high on the final differential) and high accuracy can occur with low efficiency (not thinking of a diagnosis until all suggestive data are provided but putting it high on the final differential), a third composite metric, Diagnostic Acumen Precision Performance (DAPP), was developed. It is calculated from an equally weighted average of the percentiles of both accuracy and efficiency for each solve attempt. This calculation for DAPP was conceived a priori, and it is consistent with the Institute of Medicine’s emphasis on both timely and accurate diagnosis.

### Establishing Validity Evidence for Human Dx

The research team assembled for this study has expertise in medical education, clinical excellence, diagnostic reasoning, and assessment. Furthermore, in considering the ways in which to measure and score diagnostic performance on the Human Dx platform, we presented ideas at institutional research conferences and met with consultants—both of which resulted in iterative revisions. These steps along with a comprehensive literature review confer content validity evidence to the scoring system.^11^

With respect to internal structure validity, we understood that the diagnostic performance score would need to discriminate between those who had completed training with the most clinical experience and presumably the most knowledge (attending physicians) and novices (medical students). Human Dx cases cover a broad range of areas within the concepts under study (both clinical fields and clinical reasoning strategies). Internal consistency reliability was assessed by evaluating the degree to which accuracy and efficiency, both probing aspects of diagnostic performance, would yield similar results across cases and within individuals.

The lack of other measures assessing diagnostic performance posed a challenge for establishing relationships to other variables of validity evidence. Because admission decisions at medical schools and residency programs consider performance on validated standardized exams (Medical College Admission Test and United States Medical Licensing Examination, respectively), analyses were performed to explore the associations between Human Dx scores and having affiliation with an institution ranked in the top 25 for National Institutes of Health (NIH) research funding and a medical school ranked among the top 25 by *US News and World Report* (USNWR).^12^ In general, trainees admitted to these institutions are known to have higher scores on these tests assessing knowledge.^13^

The inclusion of only highly rated GMR cases for this study, judged to be particularly clear, corroborates response process validity evidence.

### Statistical Analysis

Descriptive characteristics, including means and standard errors for the applicable variables, were computed. Linear mixed models were used to compare accuracy, efficiency, and DAPP among solvers of different levels of training (attending, resident, intern, and medical student) and affiliated academic institution (top 25 ranking for NIH grant funding or not and top 25 USNWR–ranked medical school or not), with random case and solver effects. No fixed effect other than solver tenure was adjusted. The models were fitted using restrictive maximum likelihood, and nominal *P* values were calculated using *t* statistics. For binary accuracy, we used generalized linear mixed models with logit link and random case effects. The models were fitted using pseudolikelihood, and nominal *P* values were calculated using *t* statistics. To adjust for multiple comparisons, we used the Tukey-Kramer method for pairwise comparisons among solver groups. To assess internal consistency between accuracy and efficiency we calculated the Cronbach α.^14^ The intraclass correlation coefficient (ICC) for DAPP was calculated using the ratio of the variance between solvers and the sum of the variance between solvers and the residual variance, which were estimated from a random-effects model with a random solver effect.^15^ Because the ICC for a single solve was low and not reflective of the design of the platform, we then used Spearman-Brown prophecy formula to calculate the ICC when 10 solves were averaged.^16^ The Bonferroni correction was used for analysis of the top 25 USNWR and top 25 NIH funded distinction. We used SAS statistical software version 9.3 (SAS Institute) for all analyses, and a 2-sided adjusted *P* value of less than .05 was considered statistically significant.

## Results

A total of 11 023 solves by 1738 individual solvers across 170 unique case simulations were included in the analyses. Participants included 239 attending physicians, 926 residents, 347 interns, and 226 medical students; data on sex and age are not available on Human Dx. The mean number of cases completed by each solver on the platform was 74 and the median (interquartile range) number of GMR cases completed by each solver was 2.0 (1.0-6.0). The median (interquartile range) solve time was 2.6 (1.5-4.4) minutes per case. Solver characteristics are shown in Table 1.

**Table 1.** Participant Characteristics

Experience Level	Participants, No.	Institutions Represented, No.	GMR Cases Completed, Median (IQR), No.
Attending	239	140	2.0 (1.0-6.0)
Resident	926	189	2.0 (1.0-6.0)
Intern	347	109	3.0 (1.0-7.0)
Medical student	226	70	2.0 (1.0-5.0)

Abbreviations: GMR, Global Morning Report; IQR, interquartile range.

### Accuracy Data

Interns and medical students were less likely to have the correct diagnosis listed in the first position of their differential compared with attending physicians (odds ratio [OR], 0.720; 95% CI, 0.593-0.875 and OR, 0.575; 95% CI, 0.466-0.709, respectively; *P* < .001 for both), and residents (OR, 0.720; 95% CI, 0.616-0.841 and OR, 0.574; 95% CI, 0.481-0.686, respectively; *P* < .001 for both) (Table 2). Based on the ranking of the correct diagnosis compared with all solvers of the same case, analysis of accuracy demonstrated highest mean (SE) scores for attending physicians and residents at 76.9 (1.2) and 76.8 (0.8), followed by interns at 74.7 (1.1), and medical students at 68.8 (1.3). Attending physicians had higher mean scores than medical students (difference, 8.1; 95% CI, 4.2-12.0; *P* < .001), as did residents (difference, 8.0; 95% CI, 4.8-11.2; *P* < .001) and interns (difference, 5.9; 95% CI, 2.3-9.6; *P* < .001).

**Table 2.** Participant Accuracy by Level of Training

Level of Training	Accuracy	
	Comparison With Attending, OR (95% CI)	Adjusted *P* Value
Attending	1 [Reference]	NA
Resident	1.001 (0.853-1.174)	>.99
Intern	0.720 (0.593-0.875)	<.001
Medical student	0.575 (0.466-0.709)	<.001

Abbreviations: NA, not applicable; OR, odds ratio.

### Efficiency Data

Attending physicians arrived at the correct diagnosis with less information; they had significantly higher mean scores than residents (difference, 4.8; 95% CI, 1.8-7.8; *P* < .001), interns (difference, 5.0; 95% CI, 1.5-8.4; *P* = .001), and medical students (difference, 5.4; 95% CI, 1.4-9.3; *P* = .003) (Table 3). Residents, interns, and medical students had similar efficiency performance (Table 3).

**Table 3.** Participant Efficiency and Diagnostic Acumen Precision Performance by Level of Training

Level of Training	Efficiency			Diagnostic Acumen Precision Performance		
	Percentile Score, Mean (SE)	Comparison With Attending, Mean (SE)	Adjusted *P* Value	Percentile Score, Mean (SE)	Comparison With Attending, Mean (SE)	Adjusted *P* Value
Attending	71.9 (1.2)	NA	NA	74.4 (1.0)	NA	NA
Resident	67.0 (0.7)	−4.8 (1.2)	<.001	71.8 (0.6)	−2.6 (1.0)	.05
Intern	66.9 (1.0)	−5.0 (1.4)	.001	70.8 (0.9)	−3.6 (1.2)	.01
Student	66.5 (1.2)	−5.4 (1.5)	.003	67.7 (1.1)	−6.7 (1.3)	<.001

Abbreviation: NA, not applicable.

### DAPP Score

Attending physicians had significantly higher mean DAPP scores than residents (difference, 2.6; 95% CI, 0.0-5.2; *P* = .05), interns (difference, 3.6; 95% CI, 0.6-6.6; *P* = .01), and medical students (difference, 6.7; 95% CI, 3.3-10.2; *P* < .001) (Table 3).

### Internal Consistency

The internal consistency between accuracy and efficiency using the Cronbach α was acceptable (attending: 0.688, resident: 0.644, intern: 0.623, and medical student: 0.753).^16^ The ICCs for the DAPP scores were fair to good according to conventional standards when averaged over 10 solves (attending: 0.55, resident: 0.54, intern: 0.38, and medical student: 0.67).^17^

### Association With Other Variables’ Validity Data

Forty percent of analyzed participants were affiliated with a USNWR top 25–ranked medical school (n = 496 affiliated vs 1242 nonaffiliated). Mean diagnostic percentile scores for DAPP were highest for attending physicians affiliated with a USNWR top 25 medical school compared with nonaffiliated attending physicians (80 [95% CI, 77-83] vs 72 [95% CI, 70-74], respectively; *P* < .001). Resident physicians affiliated with a USNWR top 25 medical school had higher mean DAPP scores compared with nonaffiliated peers (75 [95% CI, 73-77] vs 71 [95% CI, 69-72], respectively; *P* < .001). Interns affiliated with USNWR top 25 medical schools had higher mean DAPP scores compared with nonaffiliated peers (75 [95% CI 72-78] vs 69 [95% CI, 67-71], respectively; *P* < .001). Difference in mean DAPP scores for medical students affiliated with a USNWR top 25 medical school (69; 95% CI, 66-71) and those nonaffiliated (67; 95% CI, 64-70) did not reach statistical significance (*P* > .99). Thirty-two percent of analyzed participants were affiliated with a top 25 NIH-funded institution (n = 417 vs n = 1310. Attending physicians affiliated with a top 25 NIH-funded institution had higher mean DAPP scores compared with nonaffiliated attending physicians (81 [95% CI, 75-85] vs 72 [95% CI, 70-74], respectively; *P* < .001). Resident physicians affiliated with top 25 NIH-funded institutions had higher DAPP scores compared with nonaffiliated peers (75 [95% CI, 73-77] vs 71 [95% CI, 69-72], respectively; *P* < .001). Interns affiliated with top 25 NIH-funded institutions had higher mean DAPP scores compared with nonaffiliated peers (76 [95% CI, 73-79] vs 69 [95% CI, 67-71], respectively; *P* < .001). Difference in mean DAPP scores for medical students affiliated with a top 25 NIH-funded institution (70; 95% CI, 66-73) and those nonaffiliated (67; 95% CI, 64-69) was not statistically significant (*P* = .59).

## Discussion

Using a data set of online case simulations, we have established validity evidence for a novel measure of diagnostic performance that yields automated scoring in real time. Evaluating more than 11 023 case simulations, we found that those with more clinical experience have higher scores on 2 key components of diagnostic performance, accuracy and efficiency. Key features of these case simulations include accessibility (can be solved on a variety of online devices, including tablets, desktops, and smartphones), peer-reviewed cases, brevity (cases averaged <3 minutes to solve), computerized summative scoring of open-ended responses, and immediate feedback on performance.

The most widely used objective assessments of diagnostic performance are multiple-choice question examinations, such as United States Medical License Examination steps, Medical Council of Canada Evaluating Examination, and the American Board of Internal Medicine certifying examinations. These high-stakes summative assessments have proven validity evidence and are associated with clinically relevant practice outcomes.^18,19,20^ However, multiple-choice questions provide a predefined list of choices such that the ability to generate diagnostic considerations is not directly assessed. Another approach, key-feature problems, assesses identification of critical aspects of a variety of clinical cases including diagnosis and treatment. This method adds variability in question format and scoring, and has been used in several high-stakes assessments such as the Medical Council of Canada Qualifying Examination.^21,22^ Alternatively, script concordance tests are designed to assess the organization of clinical knowledge and the links between them; this assessment format has been shown in several populations to demonstrate higher scores among those with advanced clinical experience.^23^ While key-feature problems and script concordance tests, when well designed, provide valuable assessment of diagnostic reasoning, their development, administration, and scoring are resource and time intensive, making these approaches only reasonably well suited for high-stakes summative testing.

In contrast to the aforementioned assessments, this study uses a scalable technology designed to practically measure diagnostic performance and provide immediate feedback. New technological breakthroughs in machine learning, while largely touted for their applications to patient care, are now beginning to be used in medical education.^24,25,26^ There is real value in harnessing the experience or collective wisdom of the medical community for benchmarking and mimicking real-world practice where differentials are generated without an offering of multiple choice. Rather than selecting from a predefined list of options, these free-texted differentials are interpreted and scored by a system that uses prefixed search, autocomplete, and natural language processing to structure the data. The system then applies a machine learning algorithm created using medical ontologies, including the *International Classification of Diseases, Tenth Revision, Clinical Modification*, Unified Medical Language System, and Clinical Classifications Software, to interpret the proposed diagnoses. Thus, those entering *AAA* receive credit for accurately solving a case of abdominal aortic aneurysm. The sequential revealing of information simulates real-world patient care and clinical problem-solving conferences, while simultaneously allowing serial measurements of performance as more information becomes available.

### Limitations

Several limitations of this study should be considered. First, although poor-quality solve attempts were dropped from the data set, effort by users was likely variable; this is not unexpected as the software is currently set up as a low-stakes, self-directed learning experience. We have no reason to believe that attention to detail by a particular group, for example, students, would have been sufficiently recurrently lower to translate into a systematic bias. Second, the demographic data, including institutional affiliation, are self-described by users enrolling on Human Dx. Even though at the time of the data pull most users had been using Human Dx for less than 12 months, if a user did not update their level of training on the platform as they advanced, they could be misclassified. Third, unlike actual patient encounters where clinical information must be gathered and synthesized and a diagnosis pursued, case simulations used in our analyses provide predetermined clinical data; consequently, our assessment of diagnostic performance may not correlate with bedside diagnostic skill. Fourth, Human Dx participation is voluntary, which could result in selection bias and limit generalizability. Additionally, because GMR cases are created for teaching purposes, they often contain a pathognomonic finding as the final clue, which may explain the absence of larger differences in accuracy performance across users. Given that the Human Dx platform is virtual and automated, there may be performance bias in favor of more technologically advanced individuals. In an attempt to minimize any such biases, initial solve attempts were excluded for all users.

## Conclusions

Diagnostic acumen is paramount in providing optimal patient care. Rather than attempting to measure abstract reasoning processes, this analysis focused on concrete and actionable assessment of diagnostic performance outcomes—accuracy and efficiency in diagnosis. The online case simulations used in this analysis permitted rapid measurement of 2 critical components of diagnosis to validly assess performance. Consistent with deliberate practice, this technology provides immediate feedback based on performance, offers case-specific teaching points, and quantitatively compares performance to that of all other solvers; these features are extremely rare in medical education.^27^ The platform is currently being used by medical trainees and physicians electively as part of their self-directed learning plan; this formative assessment has the potential to assist with professional development. This advance may represent an effective step in improving diagnosis in health care through robust measurement of diagnostic performance.

## Notes

Supplement. **eAppendix.** Additional Details About The Human Diagnosis Project (Human Dx) Platform
