# Supplementary material for: Assessment of a Simulated Case-Based Measurement of Physician Diagnostic Performance
Source: JAMA Netw Open. 2019 Jan 11;2(1):e187006. doi: 10.1001/jamanetworkopen.2018.7006 (PMC6484555; doi:10.1001/jamanetworkopen.2018.7006)
Supplement: Supplement. — eAppendix. Additional Details About The Human Diagnosis Project (Human Dx) Platform [file jamanetwopen-2-e187006-s001.pdf]

Supplementary Online Content

Chatterjee S, Desai S, Manesh R, Sun J, Nundy S, Wright SM. Assessment of a simulated case-based measurement of physician diagnostic performance. *JAMA Netw Open*. 2019;2(1):e187006. doi:10.1001/jamanetworkopen.2018.7006

eAppendix. Additional Details About The Human Diagnosis Project (Human Dx) Platform

This supplementary material has been provided by the authors to give readers additional information about their work.

© 2019 Chatterjee S et al. *JAMA Network Open.*

eAppendix. Additional Details About The Human Diagnosis Project (Human Dx) Platform

The Human Diagnosis Project (Human Dx) created an intelligent training and collaboration platform enabling medical professionals to learn through engaging and interactive case-based simulations that closely mimic clinical practice and assess multiple dimensions of clinical reasoning. This platform is part of the institution's worldwide effort, created with and led by the global medical community, to build an open medical intelligence system that maps the steps to help any patient.

Similar to how anyone can contribute to online encyclopedias such as Wikipedia by authoring and editing articles, individual physicians and trainees contribute to Human Dx by authoring and solving clinical cases on the platform. As of December 2018, the Human Dx community includes over 14,000 medical professionals and trainees in over 80 countries, 500 institutions, and 40 specialties. Each user consents that their data may be used in educational research when registering.

Human Dx builds on proven methods in clinical learning by enabling medical professionals and trainees to solve these clinical cases in minutes, from any connected device, and receive immediate feedback on their clinical reasoning. These simulations reveal information sequentially, enabling users to enter and then update their differential diagnosis based on the available information. The sequential revealing of information simulates real-world patient care and Clinical Problem Solving conferences (CPSs), while simultaneously allowing serial measurements of performance as more information become available.

Human Dx's daily Global Morning Report (GMR) series provides teaching cases that are peer-reviewed by an editorial team of physician educators. Upon completing each case, learners receive teaching points and feedback. Multiple GMR series are available based on a physician's specialty and practice interest (e.g., internal medicine, family medicine, pediatrics). A brief tutorial about the Human Dx platform's case-based simulations is available at www.humandx.org/product/tutorial.

All clinical cases are authored by medical professionals either from the Human Dx user community or from its editorial team of physician experts. Clinical cases include de-identified patient data of actual patients from clinical practice and hypothetical cases designed for educational purposes (e.g., to train medical professionals on the CDC opiate prescribing guidelines). Clinical cases take less than 10 minutes to author and are automatically converted into a case-based simulation by the Human Dx system. All clinical cases are reviewed by at least one physician prior to being posted to the Human Dx platform, and a subset of clinical cases are peer-reviewed by Human Dx editors.

Human Dx uses natural language processing (NLP) and machine learning to help users enter their differential diagnoses and to score their responses. The raw data collected on each case solve includes the ranked differential diagnosis at each step of the case. As users are typing, the system identifies the most likely concepts they are thinking of by considering possible misspellings, abbreviations, and synonyms. Diagnoses are often automatically mapped to major medical ontologies including ICD-10CM and ARHQ Clinical Classification Software (CCS) but may also be free text. Human Dx uses a machine learning-based algorithm to determine when a solver's diagnosis matches the reference solution. Textual and ontological features are generated based on the relationship between the solver's diagnoses and the reference solution diagnoses. For example, string edit distance between terms and ontological distance (number of edges between the two concepts in ICD-10CM) are calculated for all pairs of diagnoses. Finally, a match is determined by a logistic regression classifier (trained on over 8,000 hand-labeled examples and 96% accurate on a held-out test set).

© 2019 Chatterjee S et al. *JAMA Network Open.*
